# Explainable artificial intelligence models for predicting pregnancy termination among reproductive-aged women in six east African countries: machine learning approach

**DOI:** 10.1186/s12884-024-06773-9

**Published:** 2024-09-16

**Authors:** Gizachew Mulu Setegn, Belayneh Endalamaw Dejene

**Affiliations:** 1 Collage of Natural and Computational Science, Department of Computer Science, Debark University, Debark, 90 Ethiopia; 2 College of Informatics, Department of Information Science, University of Gondar, Gondar, 196 Ethiopia

**Keywords:** Machine learning, Explainable Artificial Intelligence, Pregnancy termination, Reproductive-aged women, East Africa, Demographic and Health Survey

## Abstract

Pregnancy termination remains a complex and sensitive issue with approximately 45% of abortions worldwide being unsafe, and 97% of abortions occurring in developing countries. Unsafe pregnancy terminations have implications for women’s reproductive health. This research aims to compare black box models in their prediction of pregnancy termination among reproductive-aged women and identify factors associated with pregnancy termination using explainable artificial intelligence (XAI) methods. We used comprehensive secondary data on reproductive-aged women’s demographic and socioeconomic data from the Demographic Health Survey (DHS) from six countries in East Africa in the analysis. This study implemented five black box ML models, Bagging classifier, Random Forest, Extreme Gradient Boosting (XGB) Classifier, CatBoost Classifier, and Extra Trees Classifier on a dataset with 338,904 instances and 18 features. Additionally, SHAP, Eli5, and LIME XAI techniques were used to determine features associated with pregnancy termination and Statistical analysis were employed to understand the distribution of pregnancy termination. The results demonstrated that machine learning algorithms were able to predict pregnancy termination on DHS data with an overall accuracy ranging from 79.4 to 85.6%. The ML classifier random forest achieved the highest result, with an accuracy of 85.6%. Based on the results of the XAI tool, the most contributing factors for pregnancy termination are wealth index, current working experience, and source of drinking water, sex of household, education level, and marital status. The outcomes of this study using random forest is expected to significantly contribute to the field of reproductive healthcare in East Africa and can assist healthcare providers in identifying individuals’ countries at greater risk of pregnancy termination, allowing for targeted interventions and support.

## Introduction

Despite advancements in medicine, maternal mortality remains a major issue in many nations, especially developing nations [1–3]. In low- and middle-income nations, abortion ranks among the top five causes of maternal death [1]. Pregnancy termination is defined as a pregnancy that is terminated by choice through intervention, which may occur unintentionally or intentionally [1, 2]. Previous studies have suggested that unsafe pregnancy termination has a negative impact on mothers’ health and can result in abnormal birth outcomes, such as low birth weight, preterm delivery, placental issues, and abnormal birth outcomes [4–9].Adolescent and young adult abortion is a serious public health concern, particularly in developing countries.

According to estimates, there are approximately 2.2 million unplanned pregnancies and 2.5 million unsafe terminations among adolescents, and an estimated 33 abortions are carried out annually per 1,000 women between the ages of 15 and 49. The sensitive and complex issue of pregnancy termination has significant consequences for the well-being and health of women. Due to political, cultural, religious, and financial constraints, abortion during pregnancy is now a sensitive and difficult issue in reproductive health [3, 8]. In Ghana [4], descriptive and inferential binary logistic regression analyses was applied to cross sectional study data with a lack of causal interpretation of those factors due to the cross-sectional nature of the study. According to [10], in Ethiopia, using data from 15,683 women binary logistic regression, descriptive statistics were generated from a cross-sectional study of data from the EDHS. Aalmneh et al., [5] investigated the incidence of abortion and related factors among women in East Africa using DHS data from 12 East African countries were used for a sample of 431,518 women of reproductive age with a binary logistic regression [6], cross-sectional nationally representative household surveys were conducted in 36 LMICs with 1,236,330 women. Multivariable logistic regression models were employed to identify the risk factors for pregnancy termination, and 13.3% was the average pooled weighted prevalence of pregnancy termination. Healthcare prediction using artificial intelligence has become crucial in saving lives [11]. The rapid development of these systems allows for the analysis of complex data relationships to make accurate predictions in the healthcare industry. Machine learning approaches allow for the development of an accurate model that can be applied to activities like estimation, prediction, classification, or any other similar task [12–14]. This study, motivated to fill these gaps by constructing explainable artificial intelligence models, assessed the underlying structure of pregnancy termination in a sample data set monitored in East Africa There are limited studies that have determined the underlying structure, and there is a lack of a reliable and interpretable predictive model that can accurately predict pregnancy termination among reproductive-aged women in some East Africa using machine learning. We aim to fill this critical knowledge gap by developing a predictive model that combines ML techniques and XAI methods. Our main contributions to this work are as follows:

I. The underlying structure of the attributes related to pregnancy termination in a sample dataset was monitored in East Africa.
II. Different ML classifiers were evaluated using model evaluation matrix parameters to select the best algorithm for predicting pregnancy termination in East Africa.
III. XAI techniques can be used to identify major risk factors that influence the occurrence of abortion among reproductive-aged women in East Africa.

## Literature review

Several studies investigated pregnancy termination among reproductive-aged women using different methods. Djibril et al. [6] conducted a cross-sectional study to analyze 36 LMICs between 2010 and 2018. According to the study’s findings, Namibia and Pakistan had average pooled weighted prevalence rates of 13.3% and 33.4%, respectively, of pregnancy terminations. This study also found that having more than four children, being married, increasing age, and having completed elementary and secondary school were associated with pregnancy terminations using a multivariable logistic regression model. Pooled community-level pregnancy termination in East Africa was investigated by Samuel et al. [15]. Women in the age category 20–24, media exposure, being married, employment, no education, primary or secondary education, multiparty and sexual initiation of 15 or more were found to be substantially associated with termination of pregnancy based on the multilevel multivariate logistic model. Khagi et al. [16] investigated a hospital-based case-control study of the associated risk factors related to spontaneous abortion (SA) in Nepal with 252 reproductive-aged women. Descriptive statistical methodologies were conducted. The study reported that family type, folic acid intake, prenatal checkups, and monthly income were the associated risk factors for SA. The study done by Bright [2] in sub-Saharan Africa examined cross-sectional analysis with a total of 62,747 adolescent girls and young women. To investigate the factors that contribute to pregnancy termination, fixed and random effects models were employed among adolescent girls and young women. Berhanu et al. [10] used the EDHS dataset to determine the prevalence of pregnancy termination and the risk factors associated with it in women. In 7.9% of women between the ages of 15–49, education level, and awareness of the use of contraceptives were found to be strongly associated with pregnancy termination. Another study was conducted by [5]. A binary logistic regression model with a total of 431,518 women of reproductive age was used from 12 East African nations. The final model found that a higher chance of abortion was associated with media exposure, traditional family planning methods, working, being single, using tobacco or cigarettes, alcohol consumption, and a lower wealth index. However, the aforementioned studies focused on identifying determinant risk factors with a small dataset, and the majority of previous research employed descriptive statistics, which have limitations such as potentially oversimplifying complex data and accurately predicting future difficulties. Results may be hindered by descriptive analytics. Besides, these studies did not develop a predictive model, did not design the underlying structure of the attributes related to pregnancy termination, and did not apply XAI tools to understand the effect of each attribute on the prediction mode. XAI techniques can also be used to identify major risk factors that influence the occurrence of abortion among reproductive-aged women in six countries from East Africa.

## Proposed methodology

**Fig. 1:**
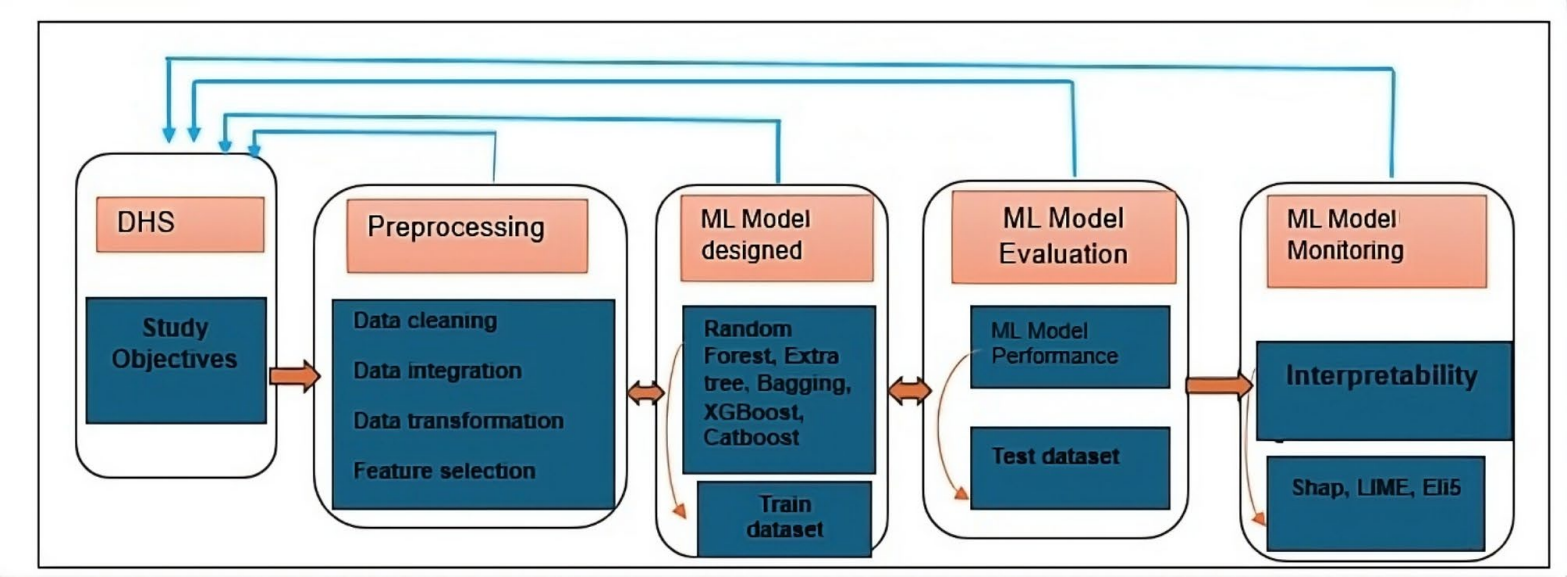
Model architecture

This study proposes a machine learning approach for the prediction of pregnancy termination among reproductive aged women with XAI tool, conducting a comprehensive and empirical analysis. The experiment consists of five steps, as illustrated in Fig. 1 the workflows and methods that were followed in this study to develop a predictive model. After analysis the study objective, data processing, model development, model evaluation and model monitoring are executed as follows:

### Data collection

The present study used data extracted from the East Africa DHS, which contained data collected from six East African countries: Ethiopia (2016), Kenya (2022), Rwanda (2019-20), Burundi (2016-17), Tanzania (2015–2016), and Uganda (2016) in five-year intervals that followed the same standard sampling procedure, questionnaires, data collection, and coding, making multicounty analysis possible and including information on pregnancies, demographic characteristics, medical history, lifestyle choices, socioeconomic factors and others (Table 1).

**Table 1:** East African DHS dataset description used for this study

No	Feature	Description
1.	Insurance coverage	Categorical (1 = Yes, 0 = No )
2.	Sex of household	Categorical (0 = Female, 1 = Male )
3.	Residence	Categorical (1 = Urban, 2 = Rural)
4.	Wanted pregnancy	Categorical (1 = Yes,0 = No )
5.	Mother Education level	Categorical (0 = No education,1 = Primary, 2 = Secondary, 3 = Higher)
6.	Household number	Continous (0 = No child,1 = one child, 2 = two child, 3 = three child….)
7.	Source of water	Continous (PIPED WATER, Public tap/standpipe, Unprotected well, protected well, Protected spring, Unprotected spring, River/dam/lake/ponds/stream/canal/irrigation channel, Rainwater…)
8.	Type of cooking	Continous (Electricity, LPG,, Natural gas, Biogas, Kerosene, lignite, Charcoal Coal, Wood, Straw/shrubs/grass, Agricultural crop, Animal dung, No food cooked in house, Others)
9.	Wealth index	Categorical (1 = Poorest, 2 = Poorer, 3 = Middle, 4 = Richer, 5 = Richest)
10.	Currently pregnancy	Categorical (1 = Yes,0 = No )
11.	Contraception use	Categorical (1 = Using modern method, 2= Using traditional method, 3 = Non-user - intends to use later, 4 = Does not intend to use 5= Never had sex)
12.	Age	Categorical (1 = 15–19, 2 = 20–24 ,3 = 25–29, 4 = 30–34 ,5 = 35–39,6 = 40–44, 7 = 45–49)
13.	Husband education	Categorical (1 = Poorest, 2 = Poorer, 3 = Middle, 4 = Richer, 5 = Richest)
14.	Frequency of watching television	Categorical (0 = Not at all,1 = Less than once a week, 2 = At least once a week,3 = Almost every day)
15.	Marital Status	Categorical (0 = Never in union,1 = Married ,2 = Living with partner,3 = Widowed, 4 = Divorced, 5 = No longer living together/separated )
16.	Currently working	Categorical (1 = Yes,0 = No )
17.	Religion	Categorical (1 = Orthodox, 2 = Catholic, 3 = Protestant ,4 = Muslin, 5 = Traditional, 6 = Other)
18.	BMI	Continous (1208, 1222,…., 5917,….)
19.	Currently breastfeeding	Categorical (1 = Yes,0 = No )
20.	Under age 18	Categorical (1 = Yes,0 = No )
21.	Smokes cigarettes	Categorical (1 = Yes,0 = No )
22.	Frequency of reading newspaper or magazine	Categorical (0 = Not at all,1 = Less than once a week, 2 = At least once a week,3 = Almost every day)
23.	Frequency of listening to radio	Categorical (0 = Not at all,1 = Less than once a week, 2 = At least once a week,3 = Almost every day)
24.	Type of mosquito bed net	Categorical (0 = No net, 1 = Only treated nets, 2 = Both treated and untreated nets, 3 = Only untreated nets)
25.	Fansidar for malaria	Categorical (1 = Yes,0 = No )
26.	Sex of child	Categorical (0 = Female, 1 = Male)
27.	Pregnancy termination as class variable	categorical (1 = Yes,0 = No )

### Data preprocessing

Before class balancing, the extracted datasets included 202,810 instances with 27 features in total including the target variable. Data preprocessing processes such as data cleaning, data transformation, and managing class imbalances were conducted. The missing values were replaced using the data imputation approach, which uses the mean for continuous data and the mode for categorical data. The process of selecting specific features for this research involves identifying a problem, reviewing existing literature, and analyzing data from the DHS. Some of the features were transformed using data discretization. In order to choose the most relevant features, we have carried out four feature selection experiments utilizing the filter (mutual information and chi-square) and wrapper (sequential forward and backward) approaches. Consequently, the step- backward feature selection technique outperforms the other methods with an accuracy of 94.7% and produces 18 important features (see Table 2). The synthetic minority oversampling technique (SMOTE) was used to balance the class without losing any important data [17].

**Table 2:** Feature selected by feature selection techniques

Feature Selection Techniques	Feature Selected	Accuracy
Mutual Information Feature Selection	‘Residence’, ‘Education level’, ‘Household number’, ‘Source of water’, ‘Sex of household’, ‘Type of cooking’, ‘Wealth index’, ‘Radio’, ‘Television’, ‘Contraception use’, Under age 18, ‘Sex of child’, ‘Husband education’, ‘Breast feeding’, ‘Martial Status’,’ Currently working’, ‘Religion’, ‘BMI’	88.0%
Chi square Feature Selection	‘Education level’, ‘Household number’, ‘Source of water’, ‘Sex of household’, ‘Wealth index’, ‘Currently pregnancy’, ‘Radio’, ‘Television’, ‘Wanted pregnancy’, ‘Insurance’, ‘Contraception use’, ‘Under age 18’, ‘Husband education’, ‘Breast feeding’, ‘Martial Status’, ‘Currently working’, ‘Religion’, ‘BMI’	89.6%
Step Backward Feature Selection	‘Education level’, ‘Household number’, ‘Source of water’,‘Sex of household’, ‘Type of cooking’, ‘Wealth index’, ‘Currently pregnancy’, ‘Radio’, ‘Television’,‘Insurance’,‘Contraception use’, Under age 18, ‘Husband education’, ‘Breast feeding’, ‘Martial Status’, ‘Currently working’,‘Religion’,‘BMI’	94.7%
Step Forward Feature Selection	‘Residence’, ‘Education level’, ‘Household number’, ‘Source of water’, ‘Type of cooking’, ‘Wealth index’, ‘Currently pregnancy’, ‘Newspaper’, ‘Wanted pregnancy, Smoking cigarattes’,‘Under age 18’, ‘Sex of child’, ‘Breast feeding’, ‘Marital Status’, ‘Martial Status’, ‘Currently working’, ‘BMI’, ‘Religion’	88.5%

### Model development

Following the completion of all necessary data preprocessing activities, a total of 338,904 instances with 18 features were considered for further analysis with ML algorithms, specifically Bagging Classifier, Random Forest (RF), XGB Classifier, CatBoost Classifier, and Extra-Trees Classifier, played a crucial part in the experiment.

### Model evaluation

Model evaluation is important for analyzing the performance of a machine learning model by using different evaluation metrics to identify strengths and weaknesses. It is crucial for assessing effectiveness in the early stages of research and for ongoing model monitoring. In this study we used Accuracy, Precision Recall, F1-score, and ROC-AUC‍ with k-fold validation. The dataset was divided into training and testing datasets at an 80/20% ratio with stratified shuffled split methods.

### Model monitoring

XAI plays a crucial role in ensuring the reliability of machine learning models. In this study, the Explainable AI algorithms SHAP, Eli5, and LIME were utilized to enhance the understanding of predictive class outcomes. This serves as a guide for planning and playing interventions for various international, continental, and national organizations to identify which region is severely affected and which further research and policy amendments are urgently needed to reduce unsafe abortion.

## Statistical analysis

The statistical analysis of the data was used to determine the underlying structure of pregnancy termination in the Eastern Africa DHS dataset by considering pregnancy type, age of reproductive women, residences, reproductive women’s country, and reproductive women’s education level. Table 3 illustrates that the prevalence of reproductive-aged women in six East Africa countries with some relevant feature only. This study on pregnancy termination distribution in East Africa reveals that women who experienced unwanted pregnancy were more likely to have an abortion. Reproductive-aged women age less than 18 years were 2.6 times more likely to have an abortion than reproductive-aged women age greater than 18 years in East Africa. One potential reason for the finding could be that younger women may have higher rates of unwanted pregnancies and abortions due to limited access to contraception and family planning services, as well as barriers such as economic resources, educational opportunities, and social support systems [18]. Women in rural areas had a higher likelihood of pregnancy termination compared to those in urban areas. A possible explanation for this finding is that women in rural areas may have restricted access to reproductive healthcare services, leading them to seek abortion as a way to avoid social stigma and exclusion related to premarital sex and pregnancy [19]. Reproductive-aged women in Uganda, Tanzania, Burundi, and Rwanda were also more likely to have an abortion. This could be explained by restricted access to modern contraceptives and reproductive health services [20]. Additionally, women with no education or only primary education had a higher history of abortion compared to those with secondary or higher education. The potential justification for this finding may be that individuals with higher levels of education typically possess greater control over their reproductive choices, leading to a better understanding of reproductive health and lower rates of unintended pregnancies and abortions compared to those with lower education levels [21].

**Table 3:** The distribution of individuals who have undergone pregnancy termination in six East Africa countries

Ever Terminated Pregnancy			
Attribute used	Feature value	Yes	No
Age of reproductive-age women	Age of reproductive-age women < 18	60%	14%
	Age of reproductive-age women > 18	20%	6%
Pregnancy Type	Wanted pregnancy	7%	1%
	Unwanted pregnancy	19%	73%
Residence of Women	Urban areas	4%	17%
	Rural areas	16%	63%
Country	Tanzania	4%	15%
	Burundi	3%	8%
	Rwanda	3%	11%
	Ethiopia	2%	17%
	Kenya	1%	8%
	Uganda	7%	20%
Education Levels of Reproductive Women	No education	7%	30%
	Primary education	11%	39%
	Secondary education	2%	9%
	Higher education	0%	2%

## Results

In this research, five ensemble ML models were used: the Bagging Classifier, Random Forest (RF), XGB Classifier, CatBoost Classifier, and Extra-Trees Classifier with the XAI tool was employed to predict and determine features associated with pregnancy termination. The data of 338,904 reproductive women were included in the dataset. The dataset used in the study initially contained 27 features and a target class that classifies the reproductive women abortion samples into Yes and No domains. For feature selection, a step-forward feature selection technique was used, and the class level of the original dataset was unbalanced; thus, the synthetic minority oversampling technique (SMOTE) was used. Three XAI techniques were used. The holdout method was used to divide the dataset into training and testing sets at a ratio of 80:20.

From Table 4 the RF model outperforms the other models in terms of accuracy in the test dataset before SMOTE analysis.

**Table 4:** Accuracy, precision, recall, F1_score, and *ROC* when performing cross-validation (k = 20) on the training and test datasets before SMOTE analysis

Algorithm	Accuracy	Precision	Recall	F1-Score	ROC
Bagging Classifier	80.2%	80.1%	80.1%	71.3%	50.0%
Random Forest	82.5%	79.4%	82.5%	77.9%	58.0%
XGB Classifier	81.1%	81.0%	81.1%	74.0%	53.0%
CatBoost Classifier	80.4%	84.2%	80.4%	71.9%	51.0%
Extra Trees Classifier	80.1%	80.1%	80.1%	80.1%	50.0%

**Fig. 2:**
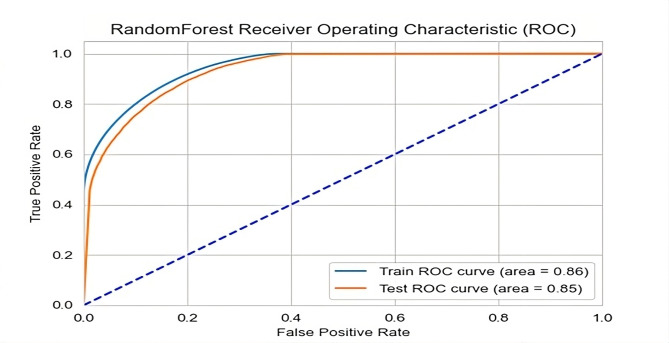
Receiver operating characteristic (ROC) curves of the RF classification models for both the training and testing datasets (model performance can be evaluated based on its false positive rate (FPR) and true positive rate (TPR). A curve that hugs the top left corner indicates a well-performing model with a low FPR and a high TPR, while points along the diagonal represent the baseline for a random classifier where FPR equals TPR)

From Table 5 the RF model outperforms the other models in terms of accuracy in the test dataset. Because of its robustness to overfitting, capacity to manage high-dimensional data with numerous features, and strong default performance without the need for considerable hyperparameter tuning, it is less susceptible to noise and outliers and increases its performance compared to other machine learning algorithms. To interpret the classification technique, we used RF for these reasons [22]. The best-performing model’s ROC curve is shown in Fig. 2. Each graph has two curves: the baseline, which is a threshold that denotes a starting point or minimum for comparisons, and the ROC curve, which shows a prediction.

**Table 5:** Accuracy, precision, recall, F1_score, and *ROC* when performing cross-validation (k = 20) on the training and test datasets after SMOTE analysis

Algorithm	Accuracy	Precision	Recall	F1-Score	ROC
Bagging Classifier	79.4%	79.5%	79.4%	79.4%	79.0%
Random Forest	85.6%	85.8%	85.6%	85.6%	85.0%
XGB Classifier	84.9%	85%	84.9%	85%	85.0%
CatBoost Classifier	80.6%	80.7%	80.7%	80.5%	81.0%
Extra Trees Classifier	84.9%	85%	84.9%	84.9%	85.0%

## Explanations of ML models

**Fig. 3:**
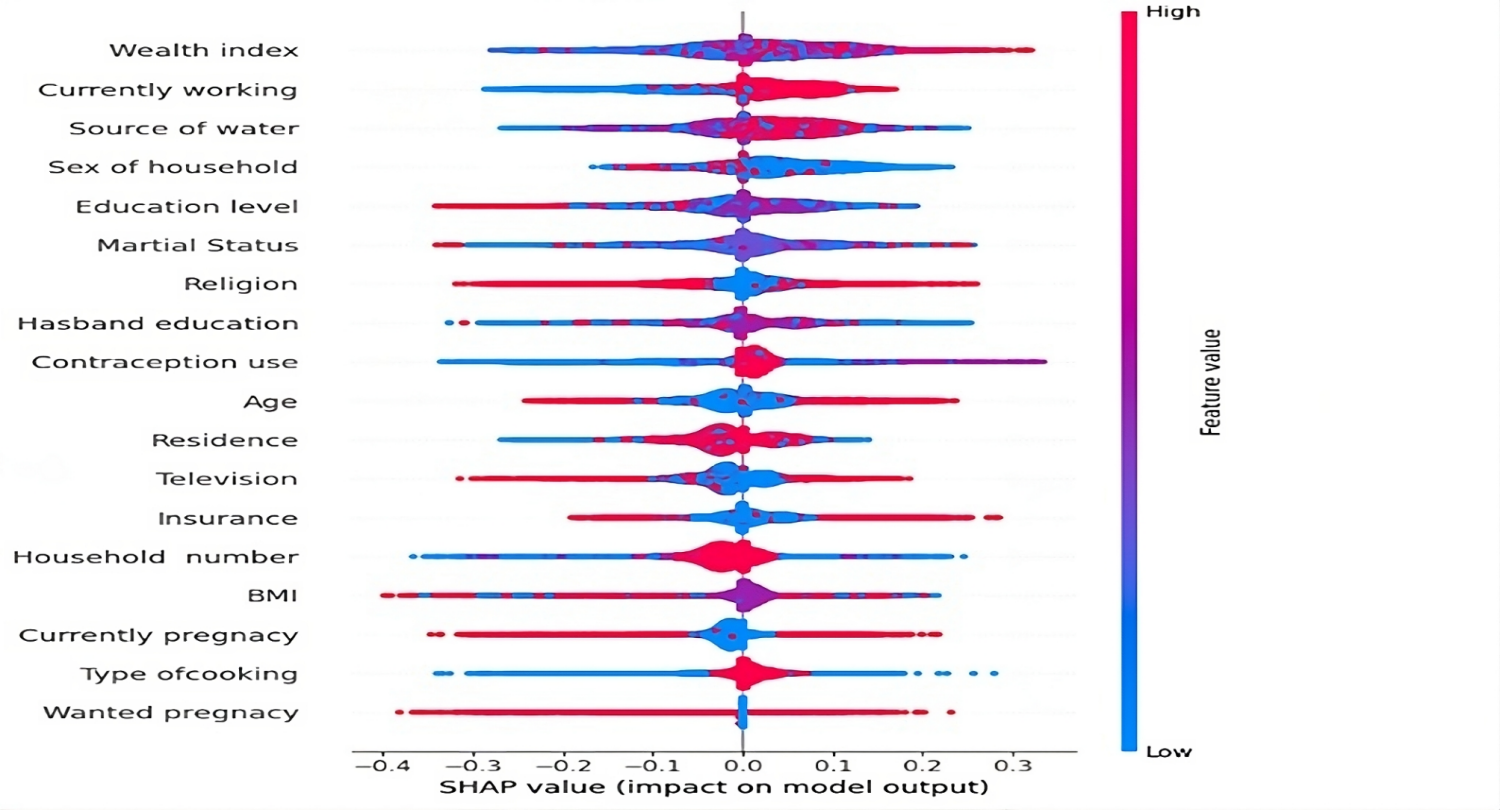
Beeswarm plot, ranked by mean absolute SHAP value (The figure uses color to show the contribution of each feature for the model, with red color representing the feature having high contribution for the model and blue color representing the feature having low contribution for the model). Figure 4 displays the results for reproductive-aged women undergoing pregnancy termination using a random forest ensemble algorithm. It is evident from the illustration that the features that are important for reproductive-aged women to terminate pregnancy are represented by red in the bar chart

XAI is currently a key focus in research to improve transparency between the working model and the user. In particular, explainable AI (XAI) techniques are essential for clarifying the reasoning behind the system’s predictions and decisions when handling sensitive health data [23]. The lack of transparency in AI and ML systems has been a persistent issue in healthcare, but the use of interpretable techniques could help to overcome this challenge [24]. In this study we used XAI tools SHAP, Eli5, and LIME to describe how attributes contributed to the prediction model. The force plot for local interpretation generated by SHAP analysis is displayed in Fig. 3. According to the figure, the wealth index, current working experience, source of drinking water, sex of the household, education level, and marital status were the main socio-demographic attributes contributing to reproductive women’s pregnancy termination.

**Fig. 4:**
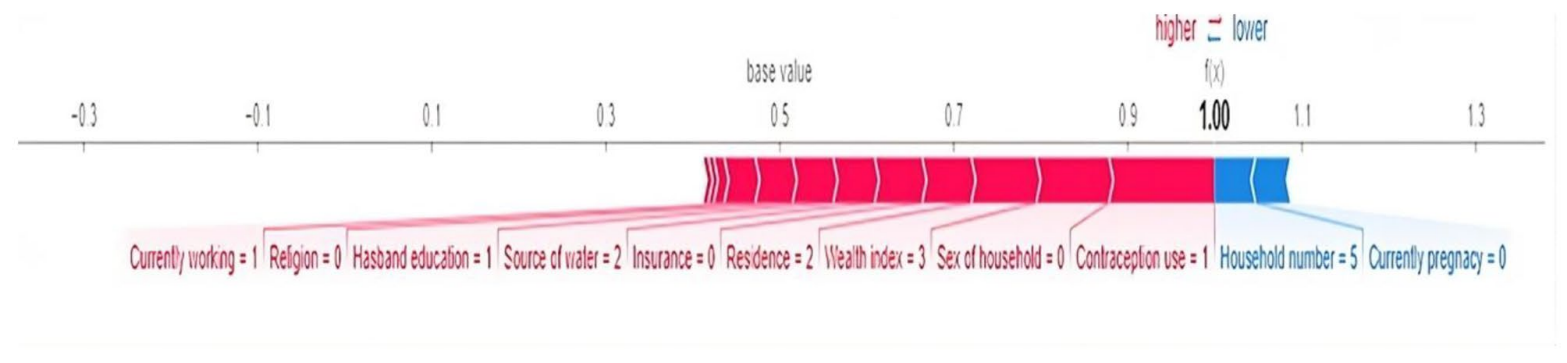
Shap interpretation. Force plot (local interpretation) for random forest algorithm and the red color in the bar chart represents the attributes that contributed to a reproductive-aged women to have a pregnancy termination

**Fig. 5:**
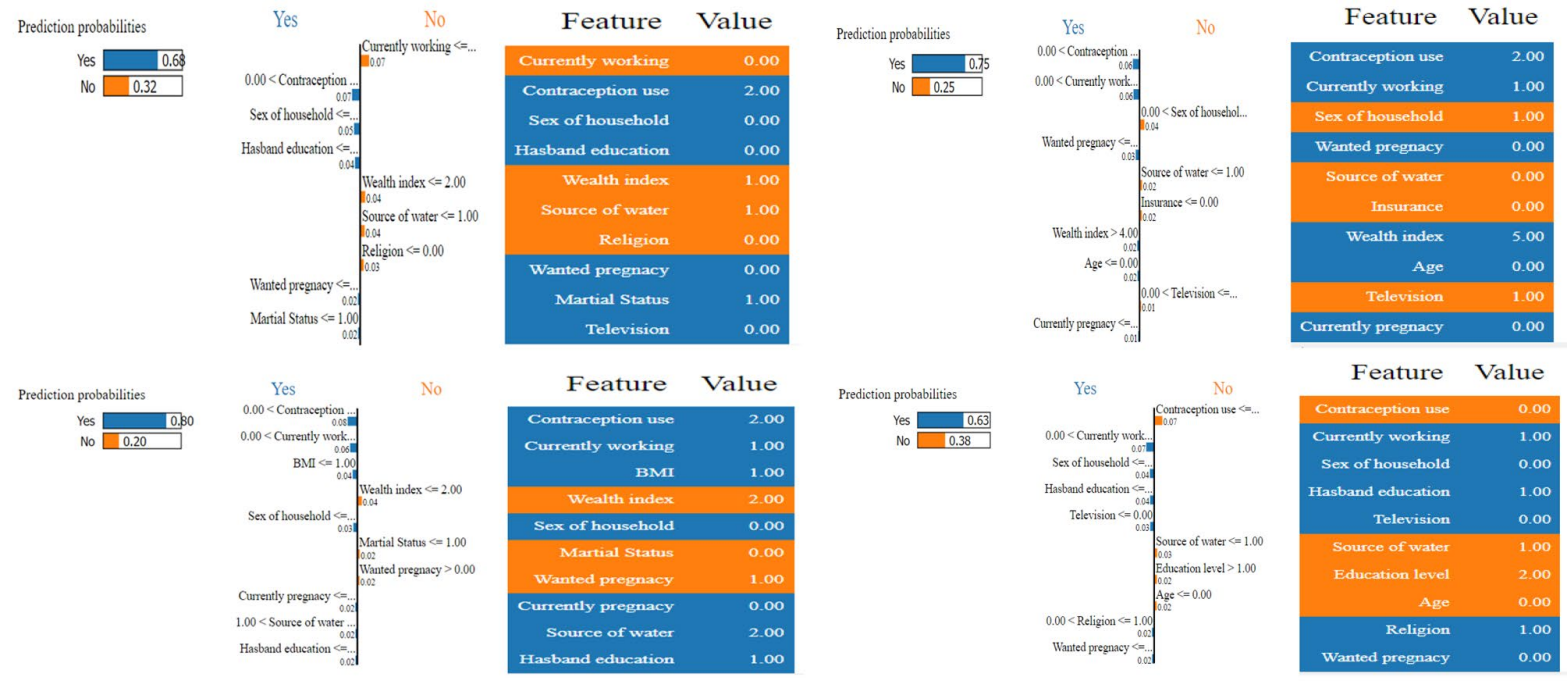
Explaining prediction using LIME

The LIME technique explains the predictions made by machine learning models by building a local, interpretable model around the prediction point [25–27]. It provides interpretable explanations locally by extracting the subset of the original data that are most important for the prediction and fitting a simple model to explain the relationship between these. Figure 5 shows the LIME visualization for the RF model to predict, for instance, the pregnancy termination class. The predicted probabilities of test pregnancy termination Yes instance and pregnancy termination No instance are 32% and 68%, respectively. The most important features of this model are the use of contraception, the source of drinking water, the husband’s education, and the education level of reproductive women. The feature importance of these features is 6%, 4%, 3%, and 2%, respectively, according to the random forest model.

As observed in Table 6, the Eli5 model utilizes RF to determine the most important local contribution for predicting a test sample as a pregnancy termination. The table’s contribution column indicates the significance. According to the Eli5 methodology, the ML models provided reproductive women’s residence, wealth index, cooking style, marital status, insurance coverage, number of households, current working experience, body mass index, media coverage on television, gender of the household, and level of education more strongly associated factors than other features.

**Table 6:** Explanation of the local contribution of features through the Eli5 model in classifying a single test instance (predicted class = pregnancy termination yes) using the RF. The importance of the features is denoted by a positive contribution

Y = 1(Probability 0.679) top Feature		
Contribution?	**Feature**	**Value**
+ 0.496	< BIAS>	1.000
+ 0.164	Residence	2.000
+ 0.085	Wealth Index	5.000
+ 0.062	Type of Cooking	2.000
+ 0.045	Marital Status	1.000
+ 0.043	Insurance	1.000
+ 0.037	Household number	5.000
+ 0.029	Currently Working	1.000
+ 0.018	Body mass Index	1.000
+ 0.017	Television	1.000
+ 0.014	Sex of Household	0.000
+ 0.009	Education Level	2.000
-0.004	Wanted Pregnancy	0.000
-0.012	Religion	1.000
-0.013	Age	0.000
-0.091	Household Education Level	2.000
-0.177	Source of Water	0.000

## Discussion

The factors that contribute to the occurrence of pregnancy termination must be recognized in order to successfully prevent maternal mortality and morbidity during pregnancy termination. Thus, wealth index, place of residence, maternal occupational status, education level, and contraceptive use among reproductive-aged women were the variables that were significant predictors of pregnancy termination. Multilevel binary logistic regression can be used to predict abortion as a preliminary decision support system [5]. The wealth index is a significant risk factor for abortion, and our findings support this finding. In Ghana [4], a cross-sectional study with 2114 datasets and binary logistic regression analyses were carried out, and age, occupational status, marital status, age at first sex, parity, place of residence, and region were identified as predictors and our findings support place of residence and occupational status. According to [10] in Ethiopia, binary logistic regression and cross-sectional studies showed that, at the 5% level of significance, pregnancy termination was strongly associated with age, education, marital status, occupation, region, and awareness of using contraceptives among 15,683 women, and our findings support education level, occupational status and contraceptive use as significant risk factors for abortion. To identify significant risk factors for abortion [16], descriptive and inferential statistical analyses (chi-square and Fisher exact tests) were used to examine the factors associated with pregnancy termination among reproductive-aged individuals. Due to the methodology (machine learning approach with XAI tool) and large number of sample size across six East Africa countries we have used the outcomes of our study have significant implications for reproductive healthcare in East Africa to identify women at risk and inform targeted interventions. The proposed model can assist healthcare providers in identifying countries with reproductive-aged women at greater risk of pregnancy termination, enabling targeted interventions and support. By leveraging ML techniques, we enhance the accuracy and robustness of the predictive model, leading to more reliable predictions.

## Conclusion

In conclusion, our study was able to use explainable machine learning models to predict pregnancy termination among reproductive-aged women on DHS data from six countries in East Africa. Wealth index, contraception method, and reproductive woman residency were found to be the most crucial variables for the prediction of pregnancy termination. Accurate prediction of pregnancy termination enable healthcare providers, policymakers, and community organizations to provide contraceptive services and post-abortion care, AI-powered predictive modeling may reduce the number of unwanted pregnancies and pregnancy terminations in low- and middle-income countries. To effectively predict individual risk, it is essential to collect comprehensive data on pregnancy termination rates within the local area and train machine learning appropriately.

### Limitations and recommendation

This study has potential limitations. As limitation, this finding may be biased due to the cross-sectional nature of the DHS data and the potential for recall bias in self-reported information on pregnancy termination, the study recognizes that it is difficult to determine causal relationships. Finally, we recommend that future researchers to incorporating additional maternal health indicators like chronic disease, and clinical dataset with model explainability techniques to predict pregnancy termination. Additionally, future research will develop artifact for end user with the help of hybrid machine learning algorithms with hyperparameter tuning and generating useful rule for policy maker and expertise for decision making in the region as well as in the world will be considered.

## Data Availability

The datasets generated and/or analyzed during the current study are available in http://dhsprogram.com/data/available-datasets.cfm

## Abbreviations

DHS: Demographic and Health Survey
EDHS: Ethiopia Demographic and Health Survey
Eli5: Explain Like I’m 5
LMICs: Low and Middle Income Countries
LIME: Local Interpretable Model-Agnostic Explanations
ML: Machine Learning
RF: Random Forest
ROC: Receiver Operating Characteristic
SHAP: Shapley Additive Explanations
SMOTE: Synthetic Minority Over-sampling Technique
WHO: World Health Organization
XAI: Explainable Artificial Intelligence
